# Unusual presentation of Sjogren’s syndrome during pregnancy: a case report

**DOI:** 10.1186/s13256-024-04563-7

**Published:** 2024-05-04

**Authors:** Vijay Sundarsingh, R. Manoj Kumar, Manjunath Kulkarni, Firas Rauf Mammoo, Pramela Renisha Rodrigues, Y. M. Prashanth

**Affiliations:** 1 Department of Critical Care Medicine, Father Muller Medical College, Mangaluru, India; 2 Department of Anaesthesiology, Father Muller Medical College, Mangaluru, India; 3 Department of Nephrology, Father Muller Medical College, Mangaluru, India; 4 Department of Internal Medicine, Father Muller Medical College, Mangaluru, India

**Keywords:** Sjogren’s syndrome (SS), Severe hypokalaemia, Renal tubular acidosis, Respiratory failure

## Abstract

**Background:**

Pregnancy imposes significant physiological changes, including alterations in electrolyte balance and renal function. This is especially important because certain disorders might worsen and make people more susceptible to electrolyte abnormalities. One such condition is Sjogren's syndrome (SS), an autoimmune disease that can cause distal renal tubular acidosis (dRTA). This case report offers a unique perspective on the intricate physiological interplay during pregnancy, emphasizing the critical importance of recognizing and managing electrolyte abnormalities, particularly in the context of autoimmune disorders such as Sjogren’s syndrome.

**Case presentation:**

We report a case of a 31-year-old pregnant Indian woman at 24 weeks gestation presenting with fever, gastrointestinal symptoms, and progressive quadriparesis followed by altered sensorium. Severe hypokalaemia and respiratory acidosis necessitated immediate intubation and ventilatory support. Investigations revealed hypokalaemia, normal anion gap metabolic acidosis, and positive autoimmune markers for SS. Concurrently, she tested positive for IgM Leptospira. Management involved aggressive correction of electrolyte imbalances and addressing the underlying SS and leptospirosis.

**Conclusion:**

This case underscores that prompt recognition and management are paramount to prevent life-threatening complications in pregnant patients with autoimmune disease. This report sheds light on the unique challenge of managing hypokalaemic quadriparesis in the context of Sjogren’s syndrome during pregnancy.

## Background

Pregnancy introduces dynamic physiological changes, including glomerular hyperfiltration and notable shifts in electrolyte balance, characterized by increased total body electrolyte stores and decreased serum levels [1]. This delicate equilibrium becomes crucial, particularly as conditions predisposing individuals to electrolyte imbalance may intensify during pregnancy, resulting in life threatening complications. Sjogren’s syndrome (SS), is an autoimmune disease affecting exocrine glands, with extra glandular manifestations. Renal involvement in SS, is documented in 18.4–67% of cases, often presents as distal renal tubular acidosis (dRTA),and is asymptomatic [2]. Hypokalemia, due to SS-related dRTA, occasionally leads to hypokalemic paralysis, resembling hypokalemic periodic paralysis [3]. This case sheds light on the intricate physiological interplay, emphasizing the importance of recognizing and managing electrolyte abnormalities, particularly during pregnancy and in the context of autoimmune disorders like Sjogren’s syndrome.

## Case report

A 31-year-old pregnant Indian woman at 24 weeks gestation presented with fever, multiple episodes of vomiting, and loose stools for 1 week. This was followed by progressive gradual onset of quadriparesis for 5 days and altered sensorium for 1 day. She never had any vaccinations in the recent past. There was no history of smoking or alcohol intake. There was no similar illness in her family. There was history of two previous caesarean sections for obstetric indications.

On arrival, her pulse rate was 130/min, BP 80/60 mm Hg, respiratory rate was 38/min, temperature was 99.8 F. Her breathing pattern was rapid and shallow. On auscultation normal vesicular breath sounds were heard, with no added sounds. Auscultation of cardiac areas was normal. Per abdominal examination revealed a gravid uterus appropriate for gestation. She was unresponsive with a GCS of E_2_V_1_M_1,_ and her pupils were dilated and fixed. In addition, she had generalized hypotonia with absent deep tendon reflexes, plantar reflexes were mute. Signs of meningism, pain, fine touch, vibration, and temperature sensation couldn’t be assessed.

Her initial arterial blood gas (ABG) analysis showed respiratory acidosis (Ph- 6.87, pCO_2_- 97 mmHg) with metabolic acidosis (HCO_3_–17.7 mEq/L) (Table 1). Her electrocardiogram showed presence of ventricular bigeminy due to severe hypokalemia (potassium–1.62 meq/L). Laboratory investigations (Table 2) showed hemoglobin of 9.2 g/dL, leukocytosis (17,100/cumm) and liver function test showed transaminitis with SGOT of 508 IU/L and SGPT–132 IU/L. Her renal function test, thyroid function tests and coagulation profile were normal.

**Table 1:** Trend of Arterial blood gas

Parameter	At admission	After 1 h of ventilation	After 24 h of ventilation
pH	6.87	7.26	7.32
pCO_2_	97 mmHg	27 mmHg	30 mmHg
pO_2_	136 mmHg	174 mmHg	121 mmHg
HCO_3_	17.7 mEq/L	12.1 mEq/L	15.3 mEq/L
Lactate	0.7 mmol/L	0.6 mmol/L	0.9 mmol/L

**Table 2:** Laboratory values at admission

Hemoglobin	9.2 g/dL	Serum bicarbonate	18.1 mEq/L
ESR	73 mm/1st hour	SGOT	508 IU/L
Total leucocyte count	17,100/cumm	SGPT	132 IU/L
Platelets	265,000/cumm	ALP	253 IU/L
Serum urea	29 mg/dL	Urine pH	7
Serum creatinine	1.08 mg/dL	Urine sodium	76 mEq/L
Serum sodium	145 mEq/L	Urine potassium	17.8 mEq/L
Serum potassium	1.62 mEq/L	Urine chloride	69 mEq/L
Serum chloride	115 mEq/L	Urinary anion gap	24.8

*ESR* Erythrocyte Sedimentation Rate, *SGOT* Serum glutamic oxaloacetic transaminase, *SGPT* Serum glutamate pyruvate transaminase, *ALP* Alkaline phosphatase

She was intubated immediately and ventilated. She was started on rapid potassium correction through a central venous catheter (initial 100 meq corrected over first 3 h). After her serum potassium levels began to improve (more than 2 mEq/L) and after appearance of sinus rhythm, she was started on intravenous bicarbonate replacements for metabolic acidosis. On further evaluation, ABG showed normal anion gap metabolic acidosis (NAGMA) with anion gap of 12, and hyperchloremia (chloride- 115 mEq/L). Urinary anion gap (UAG) was positive with a urinary Ph of 7, which suggested distal RTA. After ruling out that she was not on any medication (lithium, amphotericin B that is) causing distal RTA, an auto-immune panel was requested, which tested positive for SSA and RO-52, other auto-immune profiles for rheumatoid arthritis, SLE were negative. Post extubation, patient revealed history of foreign body sensation in the eyes and dry mouth for the past three years with a positive Schirmer’s test, suggesting Sjogren’s syndrome.

As she had fever and mild elevation of liver enzymes, she was evaluated for hepatitis A, B, C, E, malaria, dengue, which turned out to be negative. USG abdomen showed single live intra-uterine fetus with 24 weeks 5 days of gestation, normal liver texture and no features of hepatomegaly or obstruction or any intra-abdominal source of infection. She was started empirically on injection ceftriaxone 1 gm intravenous twice daily. There was no growth in blood & urine culture. The fever workup yielded a positive report for IgM Leptospira.

**Fig. 1:**
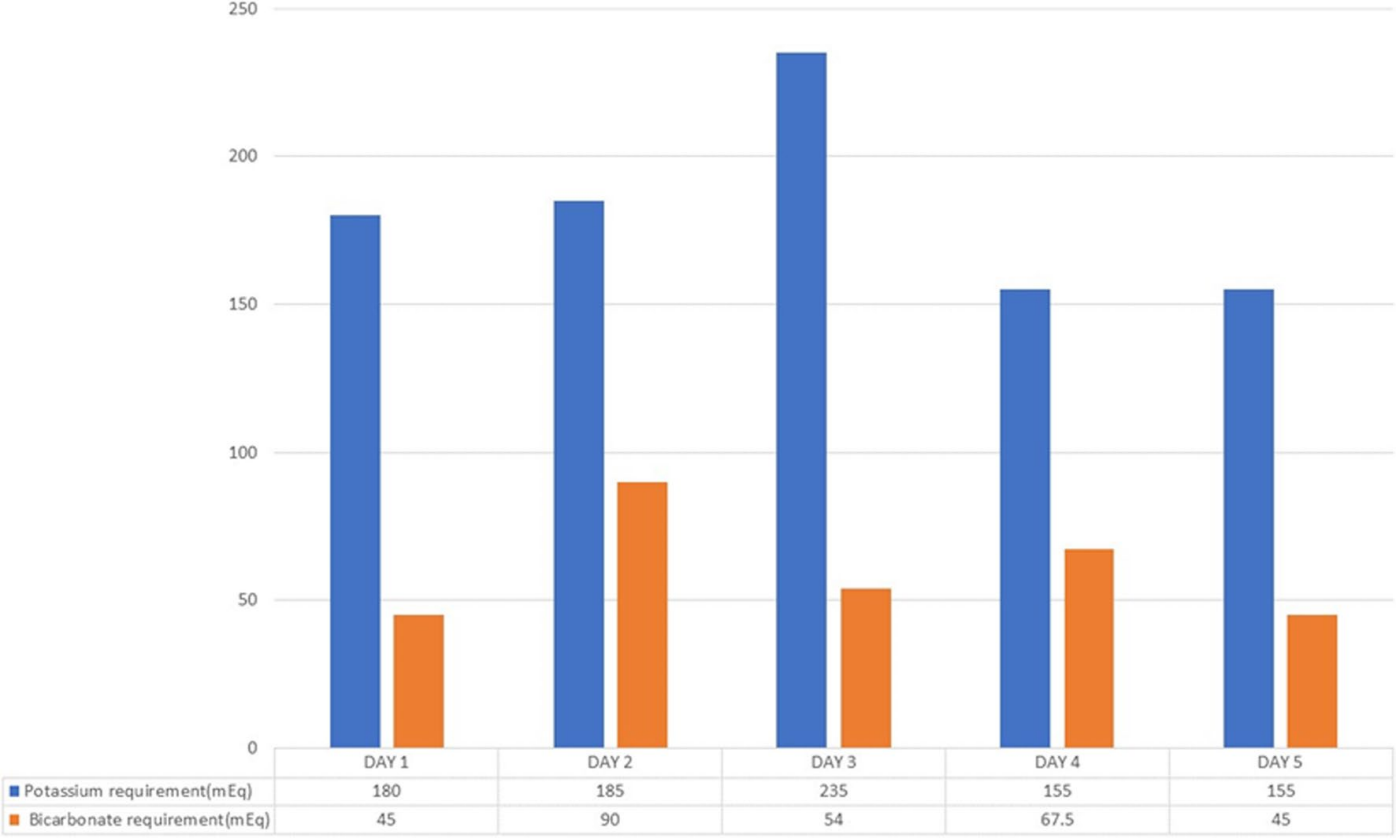
Day wise potassium and bicarbonate replacements

**Fig. 2:**
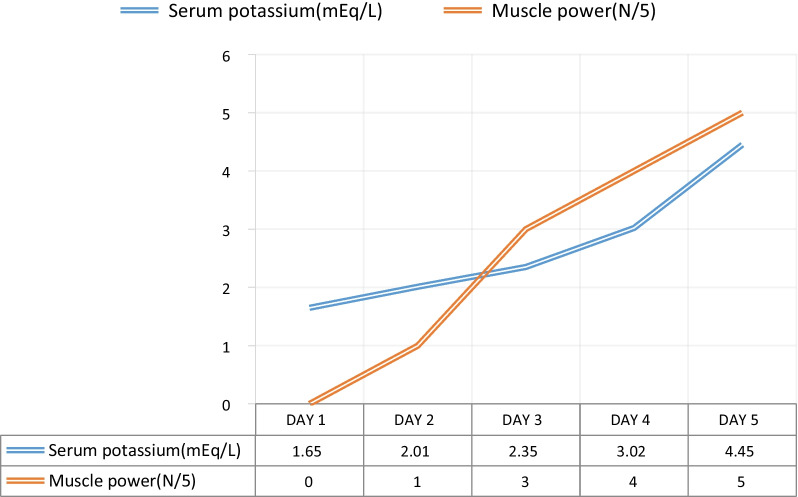
Trend of the serum potassium and muscle power (days 1–5)

**Fig. 3:**
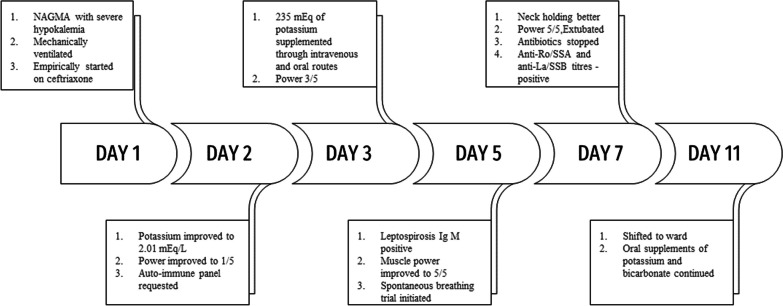
Timeline of the patient’s clinical course during her stay in hospital

For evaluation of quadriparesis, nerve conduction study was done with which showed reduced CMAP (Compound muscle action potential). During her stay in ICU fetal monitoring was done and fetus was stable throughout the stay. She required large amounts of intravenous and oral potassium replacements daily. Figure 1 shows the total potassium replacements from the admission to day 5. She regained her muscle power and started tolerating spontaneous breath trials with serial improvements in potassium (Fig. 2). She got better without any residual neuro-muscular weakness. She was extubated after a week of mechanical ventilation. MRI scan of the brain and spinal cord was planned to find the cause of quadriparesis. However, we chose not to perform the MRI because she soon began to improve after having her hypokalemia addressed. She received oral replacements of potassium and bicarbonate and was discharged. Figure 3 shows the timeline of the patient’s clinical course during her stay in hospital. She was followed up by the obstetrician and physician at regular intervals till her admission for delivery. At 36 weeks of gestation, she delivered a female baby through caesarean section.

## Discussion

Our patient presented with the complaints of muscle weakness and respiratory failure requiring mechanical ventilation and was found to have severe hypokalemia and metabolic acidosis. Patients with severe hypokalemia and metabolic acidosis are also prone to life threatening arrhythmias and should be treated emergently. Usually, metabolic acidosis is associated with hyperkalemia. A combination of metabolic acidosis and hypokalemia should raise the suspicion of renal tubular acidosis.

On further evaluation, she had normal anion gap metabolic acidosis (NAGMA), urinary pH > 5.5, and positive urinary anion gap. These biochemical findings were suggestive of the diagnosis of distal renal tubular acidosis (RTA) [2, 3]. We attributed distal RTA to Sjogren's syndrome because of history of sicca symptoms, elevated Anti-Ro/SSA antibody titres, and positive Schirmer’s test.

**Fig. 4:**
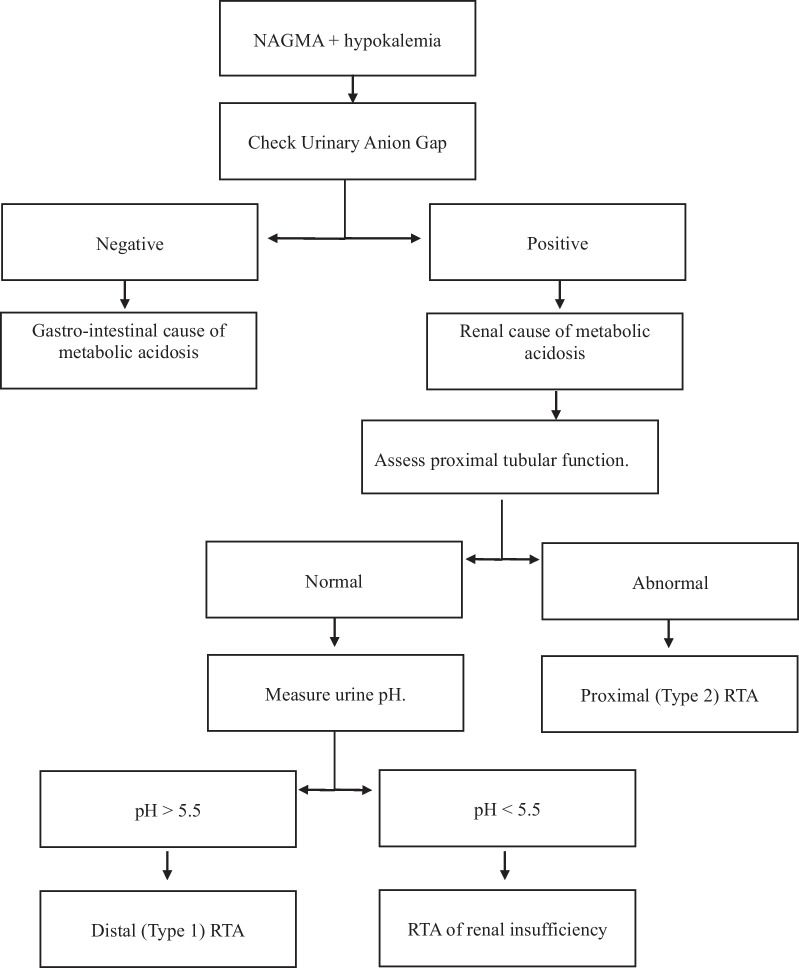
Approach to a patient with normal anion gap metabolic acidosis and hypokalemia

In patients with NAGMA and hypokalemia, urinary anion gap will help to diagnose RTA. Urinary anion gap is negative in extra-renal causes for loss of bicarbonate(eg.diarrhoea).Urinary anion gap is positive in case of renal loss of bicarbonate [4]. When urinary anion gap is positive, urinary pH and urinary electrolytes will help to diagnose the type of RTA. A normal proximal tubular function (no glucosuria / tubular proteinuria) and urinary pH greater than 5.5, confirms distal renal tubular acidosis (type 1 RTA). In RTA due to renal insufficiency, proximal tubular function is normal and urinary pH is less than 5.5. If proximal tubular function is abnormal, then it suggests proximal renal tubular acidosis (type 2 RTA). Figure 4, shows a suggested algorithmic approach for patients with NAGMA and hypokalemia.

A diagnosis of primary Sjogren’s syndrome is considered based on sicca symptoms, fatigue, and pain [5, 6]. However, systemic manifestations may sometimes provide the first clue to the disease as seen in our case, in which the presenting complaint was muscle weakness secondary to severe hypokalemia. It could be possible that channelopathies causing hypokalemic periodic paralysis was superimposed in our patient but were ruled out because they are usually associated with autosomal dominant inheritance or hyperthyroidism [7], both featuring recurrent episodes. But in our case, the patient experienced her first episode with no prior familial history of periodic paralysis, alongside normal thyroid function.

RTA secondary to Sjogren’s syndrome contributed to quadriparesis in this case [8, 9]. Approximately 5% of patients with primary Sjogren’s syndrome have clinically significant renal involvement, with distal RTA being the most common manifestation. Vaidya *et al.* also reported renal tubular acidosis-linked hypokalemic quadriparesis in an individual diagnosed with Sjogren's syndrome [10].

Management of RTA due to Sjogren’s is symptomatic. In patients with severe hypokalemia and NAGMA, the priority is to reverse the severe hypokalemia with intravenous potassium before correcting acidosis [11]. Correcting acidosis before correcting hypokalemia may lead to precipitous fall in potassium levels resulting in life threatening complications. Hence care must be taken to correct potassium to safe levels before correcting acidosis.

Our patient presented with fever, vomiting and loose stools. Fever in our patient added to the difficulty in diagnosis and management. In view of liver dysfunction, we considered differential diagnoses of infections (viral/bacterial), pre-eclampsia or eclampsia, HELLP syndrome, acute fatty liver of pregnancy hyperemesis gravidorum. Our subsequent investigations ruled out most of these. Dengue, chickungunya and leptospirosis were considered in setting of fever with weakness and hypokalemia [12, 13]. Leptospirosis was also suspected because of liver dysfunction with gastrointestinal symptoms is an endemic disease in our geographical location. Though hypokalemia is known in leptospirosis, it is usually associated with acute kidney injury [14]. However renal functions were normal in our patient, hence we considered leptospirosis as an unlikely cause for hypokalemia.

Auto-immune diseases could be asymptomatic or undiagnosed, until some infection or infection related complication aggravates [15] the symptoms. We believe leptospirosis associated vomiting might have worsened the pre-existing hypokalemia due to RTA leading to symptomatic hypokalemia in our patient.

## Conclusion

We describe a case of a pregnant woman who presented with respiratory failure and quadriplegia due to hypokalemia and RTA secondary to Sjogren’s syndrome. She was managed with ventilatory support, potassium, and bicarbonate supplements. To the best of our knowledge, this is the first case report of a pregnant woman presenting with hypokalemic paralysis, Sjogren's syndrome, and leptospirosis. This case highlights that renal tubular acidosis with hypokalemic paralysis can be a presenting feature of Sjogren's syndrome. In these patients, hypokalemia should be corrected first before correcting metabolic acidosis to prevent further worsening of hypokalemia.

## Data Availability

All case report-related information and materials are made available.
